# Clinical, biochemical, and genetic analysis of a Chinese Han pedigree with holocarboxylase synthetase deficiency: a case report

**DOI:** 10.1186/s12881-020-01080-4

**Published:** 2020-07-29

**Authors:** Zhenzhu Zheng, Gaopin Yuan, Minyan Zheng, Yiming Lin, Faming Zheng, Mengyi Jiang, Lin Zhu, Qingliu Fu

**Affiliations:** 1 Neonatal disease screening center, Quanzhou Children’s Hospital, 700 Fengze Street, Quanzhou, 362000 Fujian Province China; 2 Genuine Diagnostics Company Limited, 859 Shixiang West Road, Hangzhou, 310007 Zhejiang Province China

**Keywords:** HLCS deficiency, Case report, Arg508Trp, Frameshift mutation, Hearing damage

## Abstract

**Background:**

Holocarboxylase synthetase (HLCS) deficiency is a rare inborn disorder of biotin metabolism, which results in defects in several biotin-dependent carboxylases and presents with metabolic ketoacidosis and skin lesions.

**Case presentation:**

In this paper, we report a Chinese Han pedigree with HLCS deficiency diagnosed by using next-generation sequencing and validated with Sanger sequencing of the *HLCS* and *BTD* genes. The Chinese proband carries the common missense mutation c.1522C > T (p.Arg508Trp) in exon 9 of the *HLCS* gene, which generates an increased *K*_m_ value for biotin. A novel frameshift mutation c.1006_1007delGA (p.Glu336Thrfs*15) in exon 6 of the *HLCS* gene is predicted to be deleterious through PROVEAN and MutationTaster. A novel heterozygous mutation, c.638_642delAACAC (p.His213Profs*4), in the *BTD* gene is also identified.

**Conclusions:**

The Chinese proband carries the reported Arg508Trp variant, the novel 2-bp frameshift mutation c.1006_1007delGA (p.Glu336Thrfs*15), which expands the mutational spectrum of the *HLCS* gene, and the novel heterozygous mutation c.638_642delAACAC (p.His213Profs*4), which expands the mutational spectrum of the *BTD* gene. Furthermore, reversible hearing damage is rarely reported in patients with HLCS deficiency, which deserves further discussion.

## Background

Multiple carboxylase deficiency (MCD), a rare autosomal recessive disorder, is divided into two types depending on the pattern of enzyme deficiency: holocarboxylase synthetase deficiency (HLCS deficiency, OMIM #253270) and biotinidase deficiency (BTD, OMIM #253260). HLCS (EC 6.3.4.10) is an enzyme that catalyzes the biotinylation of four biotin-dependent carboxylases; these are pyruvate carboxylase (EC 6.4.1.1), acetyl-CoA carboxylase (EC 6.4.1.2), propionyl-CoA carboxylase (EC 6.4.1.3), and methylcrotonyl-CoA carboxylase (EC 6.4.1.4) [1, 2]. The *HLCS* gene is located on chromosome 21q22.1; pathogenic variants at this location produce severe metabolic decompensation [3]. Clinically, patients with HLCS deficiency usually present with poor feeding, respiratory distress, lethargy, vomiting, hypotonia, seizures, developmental delay, alopecia, and skin rash [4–7].

The mutation spectrum of the *HLCS* gene correlates with the clinical phenotypes, and molecular genetic analysis is helpful for definitive diagnosis [8]. An increasing number of novel pathogenic variants have been reported recently. For example, Donti et al. have revealed five cases of HLCS deficiency with broad differences in initial presentation and phenotype, and they have also described six novel pathogenic variants: c.500A > C (p.Tyr167Ser), c.1532A > T (p.Asn511Ile), c.2078G > C (p.Gly693Ala), c.977G > A (p.Gly326Glu), c.1710C > G (p.Asn570Lys), and c.1519 + 5G > A [9]. In addition, Quinonez et al. have identified the novel heterozygous variant c.996G > C (p.Gln332His) and a paracentric inversion on chromosome 21 by utilizing cytogenetic analysis [10]. In this study, we investigated a Chinese Han pedigree with HLCS deficiency and described the relationship between molecular mutation and clinical manifestation. Additionally, this HLCS deficiency patient presented the unusual clinical symptom of hearing damage during the acute episode.

## Case presentation

### Subjects

The proband was a one-year-old male patient. His nonconsanguineous parents and his elder sister were also included in this study. The patient underwent thorough physical examinations and other tests, including blood gas analysis, blood ammonia testing, plasma acylcarnitine profiling, urinary organic acid analysis, brainstem auditory evoked potential (BAEP) studies, and genetic testing. His family only underwent physical examination and genetic testing. The filter-paper dried blood spot sample was pretreated with a NeoBase Non-derivatized MS/MS Kit (PerkinElmer Life and Analytical Sciences, Turku, Finland), and the acylcarnitine profile was analyzed by using liquid chromatography–tandem mass spectrometry (Acquity TQD, Waters, Milford, MA, USA). The urinary organic acids were analyzed by using gas chromatography–tandem mass spectrometry (7890B/5977A, Agilent Technologies, Santa Clara, CA, USA). Blood gas analysis was performed with an automated blood gas analyzer (Cobas B221, Roche Diagnostics GmbH, Mannheim, Germany). The BAEP was recorded by using a KeyPoint electromyogram device (Dantec Medical A/S, Skovlunde, Denmark). Written informed consent for data collection and publication was obtained from the parents. This study was approved by the Ethical Committee, Quanzhou Children’s Hospital of Fujian. The study was prepared in accordance with the Health Insurance Portability and Accountability Act (HIPAA) regulations.

### Clinical presentation

The proband, a Chinese Han boy, was first brought to the dermatology department at the age of 1 year with a skin rash around the periorbital and perioral areas; he was treated for eczema. Three weeks later, he was referred directly to the pediatric intensive care unit with serious tachypnea, moaning, and heart failure. The rash had expanded to the limbs, neck, and groin. Capillary blood gas analysis showed metabolic acidosis with pH 6.98, a base excess of − 26 mmol/L, bicarbonate level of 3.9 mmol/L, anion gap of 25.2 mmol/L, elevated lactate of 13.1 mmol/L (normal < 2 mmol/L), and elevated ammonia of 152.0 μmol/L (normal < 47 μmol/L). Biochemical laboratory results on the day of admission showed low ornithine (15.99 μmol/L; normal 42–325 μmol/L) and a plasma acylcarnitine profile with low free carnitine and large increases in C5-OH, C5-OH/C0, C5-OH/C8, C3/C2, and C3/C0. The urinary organic acid profile displayed excessive excretion of 3-hydroxyisovaleric acid, acetylglycine, propionylglycine, 3-methylcrotonylglycine, methylcrotonylglycine, 3-hydroxybutyric acid, pyruvic acid, and lactic acid. Increases in the amounts of 2-keto-3-methyl-pentanoic acid and 2-keto-isocaproic acid suggested a metabolic disorder of the branched-chain amino acids. Based on the characteristic plasma acylcarnitine profile and urinary excretion pattern, the patient was presumptively diagnosed with MCD and treated immediately with biotin (20 mg bid). The skin rash was eliminated and the normal acid–base balance was restored 5 days later. Subsequently, the level of C5-OH decreased gradually and the urinary organic acid profile showed undetectable acetylglycine, propionylglycine, 3-methylcrotonylglycine, and methylcrotonylglycine (Table 1).

**Table 1:** Metabolites in plasma and urine

	13 months measurement	15 months measurement	17 months measurement	24 months measurement	Ref. range
**Acylcarnitine in plasma**					
C5OH (μmol/L)	3.88	1.66	0.53	0.37	0.07–0.5
C0 (μmol/L)	4.86	30.25	26.87	26.43	9.5–50
C2 (μmol/L)	7.99	9.87	11.88	10.27	3.4–45
C3 (μmol/L)	4.35	0.62	1.13	1.3	0.2–4.5
C5OH/C0	0.80	0.055	0.02	0.01	0–0.02
C5OH/C8	77.6	33.2	17.67	7.4	1.22–18
C3/C2	0.54	0.063	0.095	0.13	0.01–0.2
C3/C0	0.90	0.02	0.042	0.05	0.01–0.2
**Organic acids in urine**					
**3-methylcrotonylglycine** (μmol/mmol creatinine)	24.70	0.00	–	0.00	0
**3-hydroxyisovaleric acid** (μmol/mmol creatinine)	3.63	3.00	–	1.46	0–2.3
**methylcrotonylglycine** (μmol/mmol creatinine)	17.51	0.00	–	0.00	0
**acetylglycine** (μmol/mmol creatinine)	6.13	0.00	–	0.00	0–0.1
**propionylglycine** (μmol/mmol creatinine)	36.71	0.00	–	0.00	0
**lactic acid** (μmol/mmol creatinine)	247.95	4.23	–	4.81	0–4.7
**pyruvic acid** (μmol/mmol creatinine)	58.14	6.45	–	3.42	0–24.1
**3-hydroxybutyric acid** (μmol/mmol creatinine)	966.37	2.52	–	0.00	0–3.7
**2-keto-3-methyl pentanoic acid** (μmol/mmol creatinine)	6.16	0.00	–	0.00	0
**2-keto-isocaproic acid** (μmol/mmol creatinine)	6.16	0.00	–	0.00	0

During the acute episode, a BAEP study showed that the wave I latency and I–V interpeak latency intervals were all significantly prolonged. Additionally, the bilateral thresholds also increased: the left to 50 dbnhl and the right to 60 dbnhl. These results indicated that the patient’s bilateral hearing was impaired. The hearing impairment was predicted to be sensorineural. A cattle encephalon glycoside and ignotin injection was immediately administered. After biotin therapy for 43 days, the results of a repeat BAEP study showed that the bilateral thresholds had both decreased to 30 dbnhl, which indicated that the hearing damage had been reversed (see Fig. S1).

### DNA sequencing analysis

Peripheral whole blood or dried blood spot samples were collected from the proband and his family members. Genomic DNA was extracted by using Qiagen Blood DNA Mini Kits (Qiagen, Hilden, Germany) according to the manufacturer’s instructions and was stored at − 80 °C until further use. All exons and adjacent noncoding regions of abnormal C5-OH-related genes were amplified by polymerase chain reaction (PCR) and subsequently screened via next-generation sequencing (NGS) with a NextSeq 500/550 Buffer Cartridge v2 Sequencing Kit on a high-throughput sequencing instrument (Nextseq 500, Illumina Inc., San Diego, CA, USA). Sequence analyses were performed by using BWA, GATK, Annovar, etc.

Identified variants *HLCS* c.1522C > T, c.1006_1007delGA and *BTD* c.638_642delAACAC were validated by Sanger sequencing of samples from all family members. The *HLCS* exon 9 and exon 6 sequences were amplified by PCR using the following primers, respectively: forward 1, 5′-CTCACAGAAGCAGAACATTAT-3′ and reverse 1, 5′-GAAAACTCCGAGAGCACT-3′; forward 2, 5′-TGTAAAACGACGGCCAGTTAGTGCTATCTTTCCCCTTC-3′ and reverse 2, 5′-CAGGAAACAGCTATGACCGATGATTTCCAAACCCG-3′. *BTD* exon 4 was amplified with the following primers: forward 3, 5′-TGTAAAACGACGGCCAGTTTTAGTTGAGATGGGGTTT-3′ and reverse 3, 5′-CAGGAAACAGCTATGACCCTCCAGAGGGGTGTGTAT-3′. Sanger sequencing was performed with an ABI Prism 3500 Genetic Analyzer (Applied Biosystems, Foster City, CA, USA), and the results were analyzed with DNASTAR software (http://www.dnastar.com/).

**Fig. 1:**
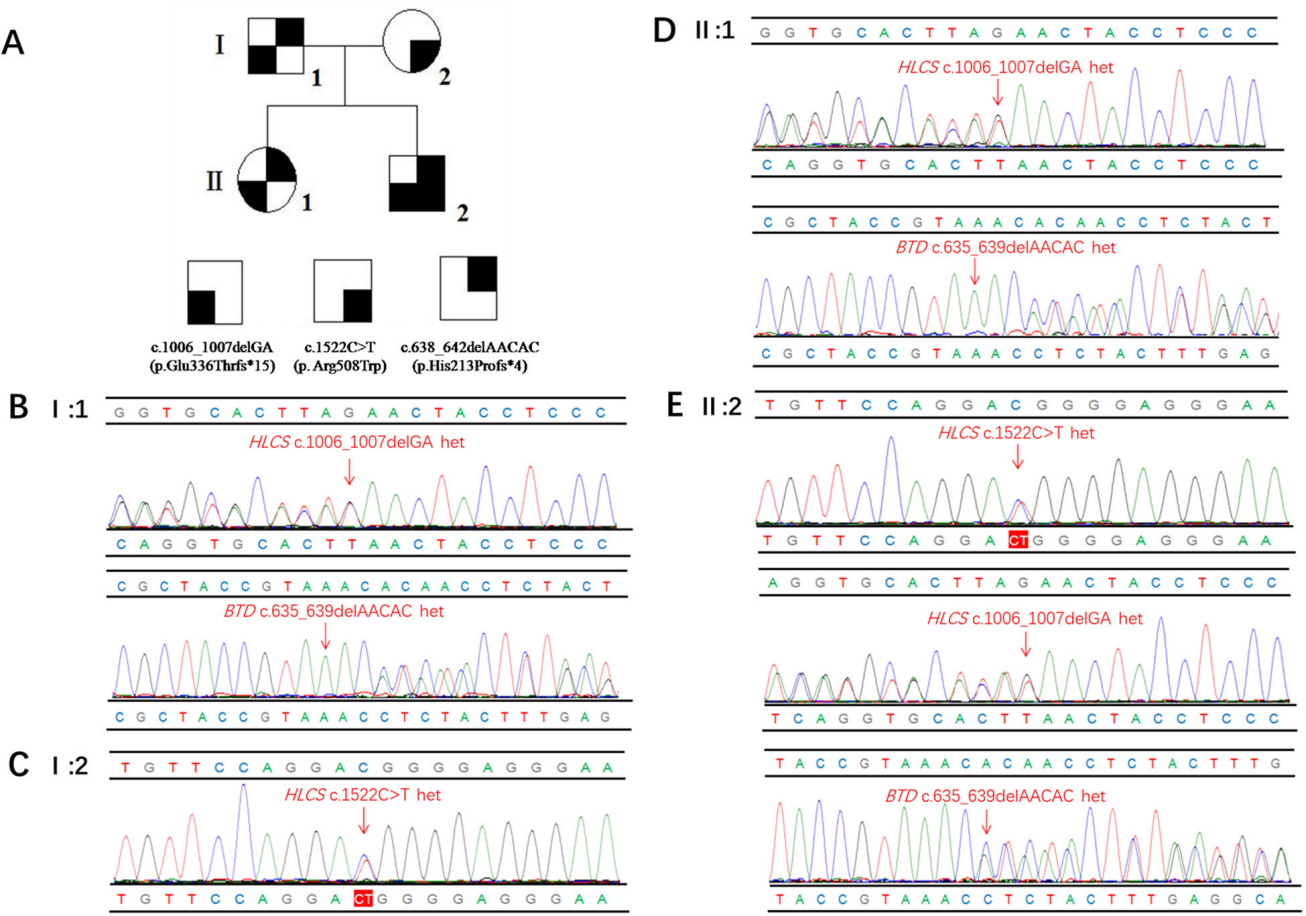
**a** Pedigree of the family. The black arrow denotes the proband. **b**-**e** Validation of the HLCS and BTD gene mutations by Sanger sequencing. Heterozygous mutations c.1522C > T, c.1006_1007delGA and c.638_642delAACAC were identified in the proband (ǁ:2), separately inherited from his father (І:1) and mother (І:2), his older sister (ǁ:1) has the same mutations with the father (the variant is indicated by a red arrow)

**Fig. 2:**
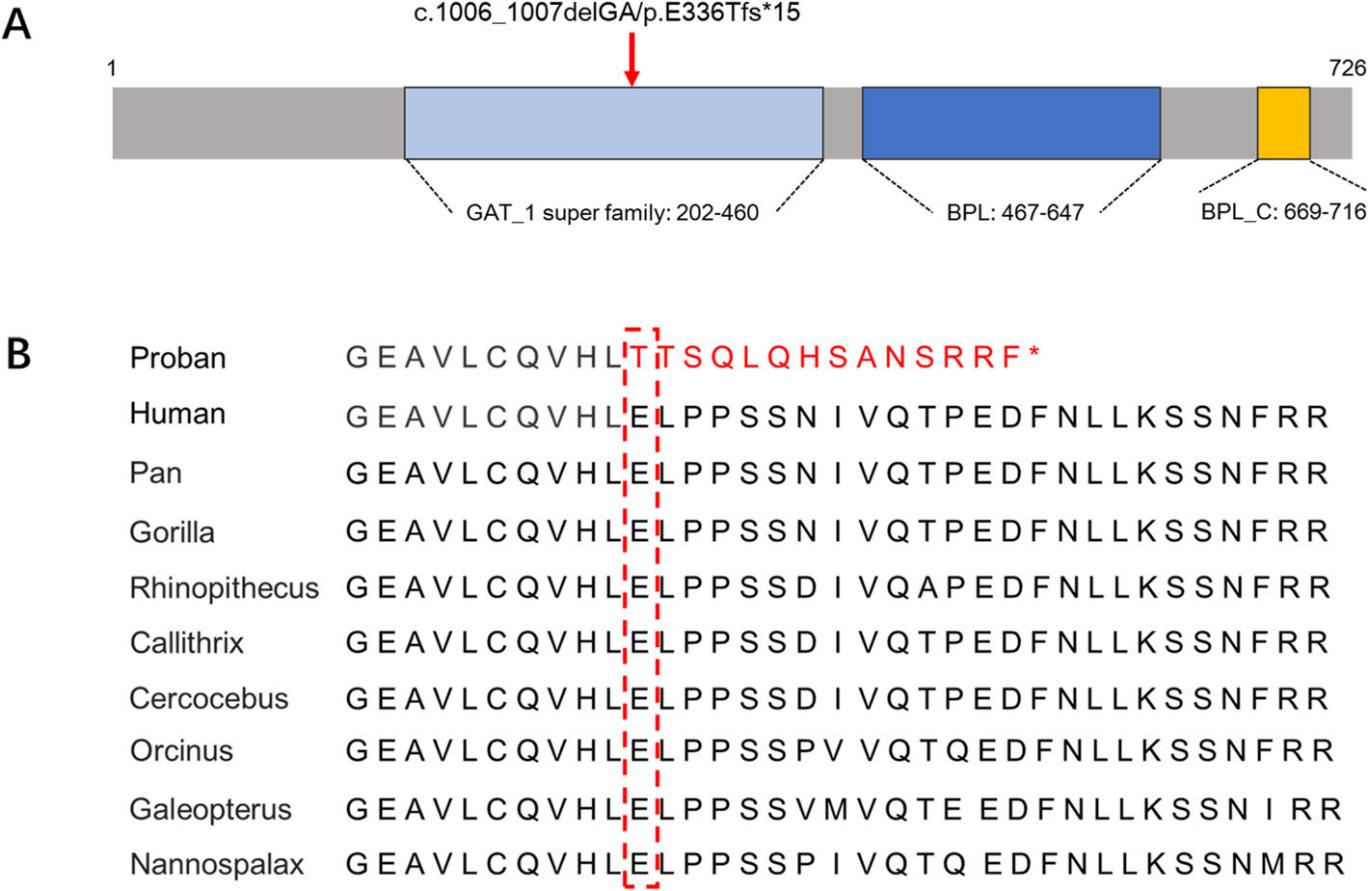
**a** Domains structure of HLCS protein. The novel frameshift mutation identified in the proband is indicated by a red arrow. **b** Conserved amino acid sequences of HLCS (amino acid 336, highlighted by a red box) and the predicted truncated HLCS caused by the frameshift mutation (c.1006_1007delGA) identified in this proband

Genetic sequencing results reveal that the patient carries the maternal missense mutation c.1522C > T (p.Arg508Trp) in exon 9 of the *HLCS* gene, the novel paternal 2-bp deletion c. 1006_1007delGA (p.Glu336Thrfs*15) in exon 6 of the *HLCS* gene, and also the novel paternal 5-bp deletion c.638_642delAACAC (p.His213Profs*4) in exon 4 of the *BTD* gene (Fig. 1e). Furthermore, his family’s genotypes have been confirmed at a heterozygous level by Sanger sequencing (Fig. 1b and c), and his healthy elder sister has been shown to carry the same mutations as his father (Fig. 1d). The pedigree is shown in Fig. 1a. The p.Arg508Trp mutation has been widely reported to be pathogenic [11, 12]. The p.Glu336Thrfs*15 and p.His213Profs*4 mutations could not be found in the literature or the 1000 Genome, ESP6500, ExAC, or dbSNP databases and were not detected in 100 healthy individuals. The effect of these mutations on protein function is predicted to be deleterious by PROVEAN and MutationTaster (Table 2). Additionally, the p.Glu336Thrfs*15 mutation resides in a conserved stretch of amino acids (Fig. 2a) and leads to truncated proteins lacking the conserved domains of HLCS (Fig. 2b). When all this information is taken into account, the c.1006_1007delGA (p.Glu336Thrfs*15) mutation is considered to be deleterious and likely pathogenic.

**Table 2:** Gene mutations in the proband with HLCS deficiency

**Gene**	*HLCS*^*A*^	*HLCS*^*A*^	*BTD*^*B*^
**Exon**	9	6	4
**Nucleotide change**	c.1522C > T	c.1006_1007delGA	c.638_642delACAAC
**Amino acid change**	p.Arg508Trp	p.Glu336Thrfs*15	p.His213Profs*4
**Parental origin**	Maternal	Paternal	Paternal
**Type of change**	Het	Het	Het
**PROVEAN/MutationTaster prediction (score)**	Deleterious(−6.559)/ Disease causing(0.99)	Deleterious(−4.224)/ Disease causing(1.0)	Deleterious(−3.927)/ Disease causing(1.0)
**ExAC MAF (Allele Count)**	0.00003302 (4/121122)	NA	NA
**Comment**	Pathogenic	Novel	Novel
**References**	Dupuis (1996) Hum Mol Genet 5, 1011 [13]	This study	This study

*Abbreviations*: *NA* not available, *Het* heterozygous, NCBI RefSeq: A, NM_000411.6; B, NM_000060.2

## Discussion and conclusions

The HLCS protein possesses three structural domains: two in the C-terminal region and one in the N-terminal half [13, 14]. It has previously been reported that most of the HLCS mutations producing increased *K*_m_ values for biotin are located in the C-terminal catalytic region [14]. Additionally, those mutations in the N-terminal region are located outside the catalytic domain and reduce the *V*_max_ of the HLCS enzyme [11].

As mentioned above, this Chinese boy carries a heterozygous c.1522C > T (p.Arg508Trp) mutation and a novel frameshift c.1006_1007delGA (p.Glu336Thrfs*15) mutation. The Arg508Trp mutation is located in the C-terminal region and generates an elevated *K*_m_ value. Patients bearing *K*_m_ mutants present the late-onset form of the deficiency and respond well to biotin therapy [15, 16]. Clinically, this patient became symptomatic at the age of 11 months, which corresponds with the late-onset form. Moreover, the novel frameshift mutation c.1006_1007delGA has been identified. This is predicted to induce an amino acid substitution of glutamate with threonine at position 336 (p.Glu336Thr). After position 336, a termination codon is formed at the fifteenth amino acid, which is predicted by PROVEAN and MutationTaster to be deleterious. The novel Glu336Thr mutation is speculated to be located outside the biotin-binding region, and the related kinetic changes require further experimental testing. However, as a result of the biotin-responsive allele (Arg508Trp), our patient can be predicted to be responsive to biotin administration [17]; the clinical outcome supports this deduction.

The BAEP study showed that the patient’s bilateral hearing had been slightly impaired. Patients with HLCS deficiency presenting abnormal BAEPs have rarely been reported, and the reason is worthy of discussion. Slavin et al. reported a girl with HLCS deficiency presenting conductive hearing loss as a result of cerumen impaction [7], but this is different from the situation with our patient. Additionally, our patient’s hearing impairment recovered after 43 days of biotin therapy, which is different from the irreversible hearing loss of biotinidase deficiency. Hence, we speculate that the hearing impairment may be secondary to the primary disorder of HLCS deficiency. However, this is just a hypothesis because there is no literature or experimental data. In any case, there are almost no reports that patients with HLCS deficiency present hearing impairment, so we believe that this case is noteworthy and the cause of the impairment needs to be confirmed.

In conclusion, we report a Chinese Han boy who shows clinical features and biochemical parameters that are consistent with the genetic data and who clearly presents HLCS deficiency. He carries a common Arg508Trp variant, which corresponds with the biotin-responsive and late-onset presentation. The novel 2-bp frameshift mutation c.1006_1007delGA (p.Glu336Thr) would expand the mutational spectrum of the *HLCS* gene. Moreover, there is a rare association of bilateral hearing damage with HLCS deficiency in this patient. However, this occurrence needs to be investigated further.

## Supplementary information

**Additional file 1: Figure S1.** A The BAEP study during an acute episode in the patient. (B) The BAEP study after biotin therapy for 43 days.

## Data Availability

The reference sequence for validation of the Arg508Trp and Glu336Thrfs*15 variants in the *HLCS* gene was acquired from the NCBI Nucleotide database by using accession number NM_000411.6. The reference sequence for validation of the His213Profs*4 variant in the *BTD* gene was acquired from the NCBI Nucleotide database by using accession number NM_000060.2. The raw sequencing data is available in the NCBI’s Sequence Read Archive (SRA) [with accession number: SRR11965409 (https://dataview.ncbi.nlm.nih.gov/object/SRR11965409)].

## Abbreviations

MCD: Multiple carboxylase deficiency
HLCS: Holocarboxylase synthetase
BTD: Biotinidase deficiency
BAEP: Brainstem auditory evoked potential
HIPAA: Health Insurance Portability and Accountability Act
PCR: Polymerase chain reaction
NGS: Next-generation sequencing
