# Supplementary material for: Clinical, biochemical, and genetic analysis of a Chinese Han pedigree with holocarboxylase synthetase deficiency: a case report
Source: BMC Med Genet. 2020 Jul 29;21:155. doi: 10.1186/s12881-020-01080-4 (PMC7388215; doi:10.1186/s12881-020-01080-4)
Supplement: Supplementary file 1 — Additional file 1: Figure S1. A The BAEP study during an acute episode in the patient. (B) The BAEP study after biotin therapy for 43 days. [file 12881_2020_1080_MOESM1_ESM.docx]

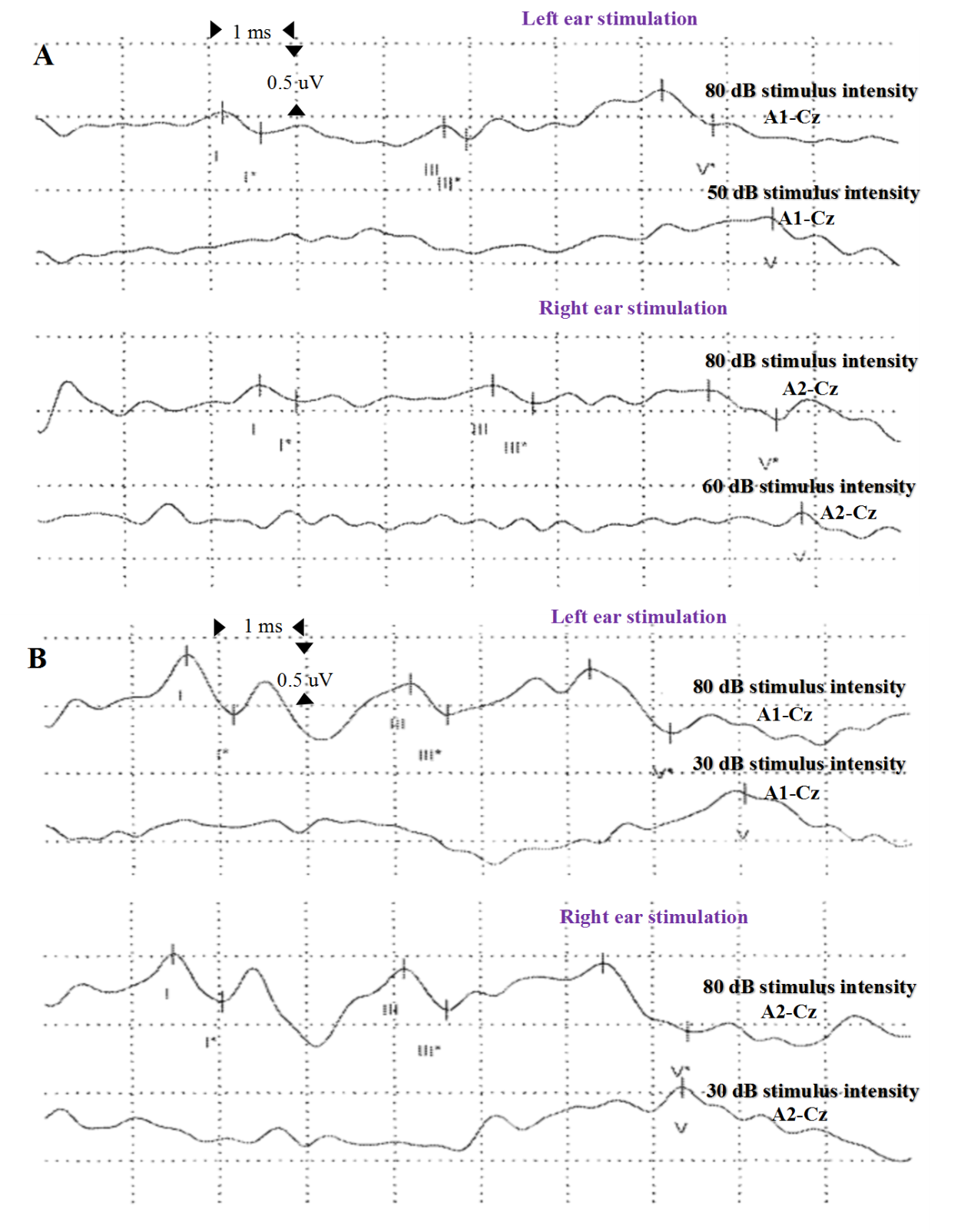


**Fig. S1** (A) A BAEP study during acute episode of the patient. (B) A BAEP study after biotin therapy for 43 days.
